# Creatine and creatinine quantification in olympic athletes: dried blood spot analysis pilot study

**DOI:** 10.5114/biolsport.2022.108701

**Published:** 2021-09-30

**Authors:** Flavio I. Bachini, Danilo Pereira, Ruan Santos, Matheus Hausen, Glauber Pereira, Camila Vieira, Lee Taylor, Marcelle Pegurier

**Affiliations:** 1 Olympic Laboratory, Brazil Olympic Committee, Rio de Janeiro, RJ, Brazil; 2 Waters Brasil, Barueri, SP, Brazil; 3 Loughborough University, School of Sport, Exercise and Health Sciences, Loughborough, UK; 4 University of Technology Sydney (UTS), Sport & Exercise Discipline Group, Faculty of Health, Australia; 5 University of Technology Sydney (UTS), Human Performance Research Centre, Australia

**Keywords:** Tandem mass spectrometry, Capillary blood, High performance athlete, Serum, FIA – MS

## Abstract

Capillary dried blood spot (DBS) samples facilitate field-based collection without venipuncture. This pilot study aims to evaluate the viability of creatine (Cr) and creatinine (Crt) quantification using fresh capillary serum (Cr_S_/Crt_S_) and DBS samples (Cr_DBS_/Crt_DBS_), using Flow Injection Analysis Mass Spectrometry (FIA – MS). Nine Olympic Athletes provided a capillary blood sample to assess Cr_S_/Crt_S_ and Cr_DBS_/Crt_DBS_ quantified by FIA – MS. No difference between Crt_S_ (mean ± SD: 813.6 ± 102.4 μmol/L) and Crt_DBS_ (812.4 ± 108.1 *μ*mol/L) was observed with acceptable variance [SEM 88.7; CV 10.7%; ICC 0.57 (CI 95% 0.06 – 0.84)] and agreement [very strong (Spearman: *r* = 0.77; *p* < 0.01) or strong (Pearson: *r* = 0.56; *p* = 0.04); Bland Altman: lower (-193) and upper (+196) limits of agreement]. Cr_S_ (mean ± SD: 691.8 ± 165.2 *μ*mol/L) was significantly different to Cr_DBS_ (2911 ± 571.4 *μ*mol/L) with unacceptable variance [SEM 171.6; CV 27%; ICC 0.002 (CI 95% -0.02 – 0.07)] and ‘weak’ agreement [Spearman: *r* = 0.21, *p* = 0.47 and Pearson: *r* = 0.06, *p* = 0.84; Bland Altman lower (-3367) and upper (-1072) limits of agreement]. Crt quantification is viable using both Crt_S_ and Crt_DBS_ (but not for Cr and Cr_S_/Cr_DBS_), with the DBS tissue handling technique offering several methodological and practice facing advantages. Future work should expand upon the sample size, explore sport/discipline relevant analytes across a full competitive season, including key training, recovery and performance blocks of their periodized performance plan.

## INTRODUCTION

**FIG. 1:**
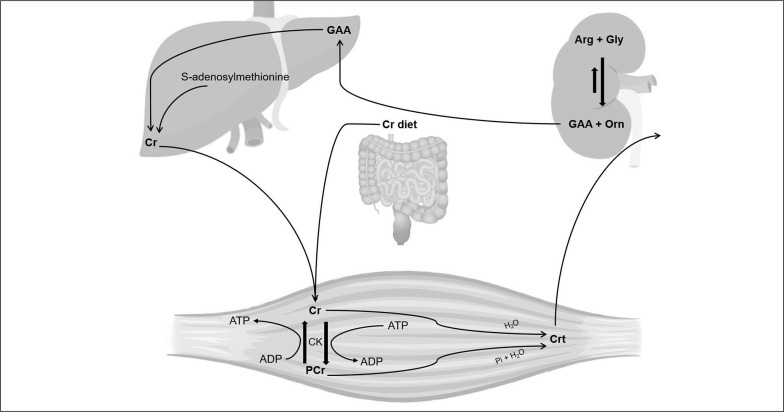
**Creatine (Cr) and creatinine (Crt) metabolites: an interrelated metabolism.** Cr dietary intake occurs either intestinal absorption or Cr biosynthesis. Cr biosynthesis depends on glycine and arginine resulting in ornithine and guanidinoacetate (GAA) within the kidney. GAA is catalyzed by N-guanidinoacetate methyltransferase and metabolized to Cr in liver. Conversion of Cr to creatine phosphate results in Crt in skeletal muscle. Cr and Crt metabolism fuel rapid ATP formation during intense metabolic demand, such as repeated skeletal muscle contraction.

The biochemical pathway of creatine (Cr) synthesis and its uses for energy suply, together with the final serum/plasma creatinine (Crt) buildup is already well documented (Figure 1). Cr/Crt metabolism is co-responsible to maintain the adenosine triphosphate (ATP) equilibrium during muscle contraction and cell hemostasis [1]. Cr supplementation increases skeletal muscle Cr and is popular with athletes (15–40%) [2]. Unsurprising, given the implication of Cr in supporting favorable adaptation to physical training, ergogenic performance effects and recovery promotion [2, 3]. Measurement of muscle injury biomarkers and neurohormones is common within elite sport practice to assess athletes training and performance, including overreaching/overtraining syndrome (OR/OTS) paradigms [4, 5]. Disruption to homeostasis due to acute or chronic exercise can alter blood catecholamines, glucocorticoids, testosterone, amino acids, as well as eliciting glycogen depletion, oxidative stress and an immune response [4]. However, there is ongoing conjecture regarding their ability to evidence-informing athlete preparedness for training/competition (e.g., logistical and practical challenges with acquiring and processing the requisite tissue, particularly in the field) [4, 6]. Cr and Crt have promise within OTS and renal stress paradigms and may offer utility relative to athlete (including Olympic) preparedness decisions [2].

Whilst this pilot study does not seek to validate for what reason Cr/Crt should be measured in athletes, it goals to use these analytes to explore whether capillary derived dried blood spot (DBS; 10–20 *μ*L/spot) compared to fresh serum tissue handling, can produce agreeable quantitative results with acceptable variance. DBS samples are field-testing-compatible and offer stability under ambient conditions for long periods without deterioration in sample accuracy and can be transported in envelopes without the need for refrigeration [7]. To quantify Cr and Crt, Flow Injection Analysis – Mass Spectrometry (FIA–MS) has the advantage of not requiring a chromatographic column while reducing acquisition time and sample/reagent quantity [8]. Therefore, if the DBS-FIA-MS (Cr_DBS_/Crt_DBS_) has acceptable results compared to fresh-capillary serum-FIA-MS (Cr_S_/Crt_S_), sample acquisition/tissue handling and subsequent laboratory analysis would be quicker and easier with greater ecological validity to practice (i.e. greater field compatibility). Essentially, Cr_DBS_/Crt_DBS_ would have enhanced utility within often time-poor elite athlete preparation, competition, and recovery paradigms, compared to Cr_S_/Crt_S_.

This short communication aims to determine the agreement and viability between the Cr_S_/Cr_DBS_ and Crt_S_/Crt_DBS_ tissue handling and quantification methods using an Olympic athlete cohort. It is hypothesized that the Cr_S_/Cr_DBS_ and Crt_S_/Crt_DBS_ methods will demonstrate acceptable agreement, error, and variance.

## MATERIALS AND METHODS

### Subjects

This is an observational study. Nine Olympic athletes [Male (7)/Female (2), Swimming (n = 01; five samples), Surf (n = 01; two samples), Volleyball (n = 06; six samples) and Race Walking (n = 01; one sample); resulting in 14 samples] provided signed informed consent prior to data collection, in accordance to the National Health Council Resolution (2012) and Declaration of Helsinki. Ethical approval was provided by the Ethics Committee of the Rio de Janeiro Municipal Health Department (protocol number 96949518.1. 0000.5279).

### Methods

#### Serum and DBS Samples

Sample was collected using contact-activated lancets BD Microtainer® (BD, Franklin Lakes, NJ, USA), blade size 2.0 mm × 1.5 mm, at the same time in the morning, following an overnight fast from participants during scheduled performance benchmarking or health screening visits to the Olympic Laboratory. The sample was processed to either: (i) immediately provide fresh serum (Crs; Crt_S_) or (ii) using DBS techniques (Cr_DBS;_ Crt_DBS_). Samples were analyzed in technical and biological replicates. Technical replicates (i.e., repeated quantification of the same sample) quantify the presence of methodological/technical error (i.e., variance) whereas biological replicates (i.e., biologically distinct samples) quantify biological variation.

##### Serum

Capillary blood (1.5mL) was collected and added into a 1.5 mL microtube (MaxyClear Snaplock, Axygen Inc., USA), allowed to rest for 60 minutes at room temperature before centrifugation (Heraeus Fresco 17 Microcentrifuge, Thermo Fisher Scientific Inc., USA) at 2,000 rpm for 10 minutes. All capillary serum obtained was isolated and added into a 2 mL Cryogenic Storage Vial (Fisher Scientific International, Inc., USA). Samples were prepared in 2 mL microcentrifuge tubes (MaxyClear Capless, Axygen Inc., USA) by adding 30 *μ*L of capillary serum to an isotopically labeled Cr/Crt standard solution (Cambridge Isotope. Inc., USA) diluted in methanol (1 mg/L). Samples were vortexed for one minute at 1,500 rpm (Digital Vortex Mixer, Fisher Scientific International, Inc., USA) and then centrifuged at 12,000 rpm for five minutes. Resulting supernatant was aliquoted to liquid chromatography–mass spectrometry certified vials [Capped Cap with PTFE/Silicone Septum, 1 mL (Waters. USA)].

##### DBS

Fresh capillary blood was dripped directly from the fingertip onto DBS specific filter paper (Whatman 903 Protein saver card, Merck, USA) until saturated and left to dry at room temperature for three hours. Subsequently, a 3.2 mm diameter, medially located circle was removed [using a sterile hand operated punch (Model number 23517097J, Fiskars, Taiwan, CHN)] and placed into a 2.0 mL Microcentrifuge Tube and then handled as per serum section.

#### Mass Spectrometry Methods

Mass Spectrometry data were acquired using a Triple Quadrupole Mass Spectrometer (Xevo^®^ TQ-S, Waters, USA) in an Electrospray Ionization Positive Mode (ES+). Mass transitions for Cr and Crt were monitored in Multiple Reaction Monitoring (MRM) mode: (i) Cr – 132.10 > 43.90 (Collision Energy 16 V); 132.10 > 89.90 (Collision Energy 11 V); (ii) Cr-d3 – 135.10 > 47.00 (Collison Energy 15 V), using 40 V of cone energy; (iii) Crt – 114.10 > 44.00 (Collision Energy 12 V); and (iv) Crt-d3 – 117.10 > 47.00 (Collision Energy 15 V), using 50 V of cone energy. The retention window utilized was 0.05/0.75 in a span of 0.1. Capillary voltage was set at 0.50 kV. Source and desolvation temperatures were 150°C and 600°C, respectively. Desolvation and cone gas flow was 900 L/Hr and 150 L/Hr. The separation was performed with an isocratic gradient flow (500 *μ*L.min-1), using ultra-pure water with 0.1% formic acid for phase A and pure acetonitrile with 0.1% of formic acid for phase B. The elution gradient was programmed as the following: 0 minute: 0.02 mL/min; 0.30 minute: 0.020 mL/min; 0.31 minute: 0.5 mL/min; 0.70 minute: 0.5 mL/min; 0.71 minute: 0.020 mL/min; and 1.00 minute: 0.020 mL/min remained until the end of the injection, using Phase A and Phase B (20:80). The samples tray temperature was controlled at 8°C during the measurement. After acquisitions, Cr and Crt were quantified using TargetLynx Software from Mass Lynx software (Waters, UK).

### Statistical Analysis

Shapiro-Wilk and Q-Q plots were used to test the assumption of normality and deemed plausible. Paired sample t-tests were performed to compare Cr_DBS_/Cr_S_ and Crt_DBS_/Crt_S_ means. Hedges’ *g* was calculated for T-test effect size, and classified as ‘trivial’ (g < 0.10), ‘small’ (0.10 ≤ g ≤ 0.29), ‘moderate’ (0.30 ≤ *g* ≤ 0.49), ‘large’ (0.50 ≤ *g* ≤ 0.69), ‘very large’ (0.70 ≤ *g* ≤ 0.89) or ‘extremely large’ (*g* ≥ 0.90) [9]. Standard error of measurement (SEM) was derived from regression line interpretation, with coefficients of variation (CV) calculated to assess absolute agreement. Limits of agreement from Cr_DBS_/Cr_S_ and Crt_DBS_/Crt_S_ means were plotted using Bland Altman [10]. Intraclass correlation [ICC; (3,1 type – Two-way mixed effects, absolute agreement, single measurements)] were calculated to evaluate the relative agreement and was interpreted as ‘small’ if ICC < 0.50; ‘moderate’ if 0.5 < ICC < 0.75; ‘good’ if 0.75 < ICC < 0.90; or ‘excellent’ if ICC > 0.90 [11] . Spearman and Pearson correlations were calculated from Cr and Crt biological means, being interpreted as: non-important (0 ≤ *r* ≤ 0.20); weak (0.20 < *r* ≤ 0.50); strong (0.50 < *r* ≤ 0.70); very strong (*r* > 0.70) [12]. Two-tailed alpha was set at *p* ≤ 0.05.

## RESULTS

**FIG. 2:**
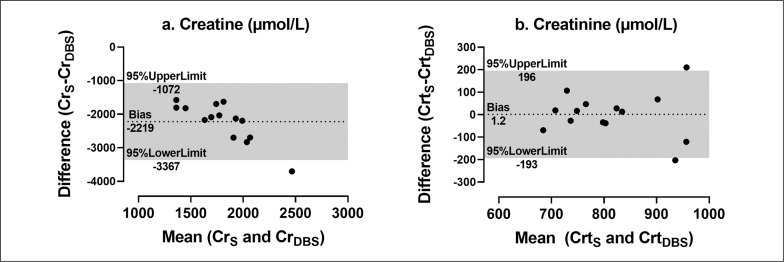
**Bland Altman plot of Creatine (Fig. 2a) and creatinine Fig. 2b) concentrations from serum and DBS samples**. **A:** Cr concentrations (μmol/L); X: Mean of Cr_S_ and Cr_DBS_ (μmol/L); Y: Mean: -2219; Lower limit: -3367; Upper limit: -1072. **B:** Crt concentrations (μmol/L); X: Mean of Crt_S_ and Crt_DBS_ (μmol/L); Y: Mean: 1.2; Lower limit: -193; Upper limit: 196.

Differences between Crt_S_ (mean ± SD: 813.6 ± 102.4 *μ*mol/L) and Crt_DBS_ (812.4 ± 108.1 *μ*mol/L) were not evident (*p* > 0.05; *g*: ‘trivial’), but a significant difference between Cr_S_ (mean ± SD: 691.8 ± 165.2 μmol/L) and Cr_DBS_ (2911 ± 571.4 μmol/L) was (*p* < 0.001; *g*: ‘extremely large’). SEM 88.7; CV 10.7%; ICC 0.57 (CI 95% 0.06 – 0.84)] and agreement [very strong (Spearman: r = 0.77; p < 0.01) or strong (Pearson: *r* = 0.56; *p* = 0.04); Bland Altman: lower (-193) and upper (+196) limits of agreement all acceptable for Crt_S_/Crt_DBS_. SEM 171.6; CV 27%; ICC 0.002 (CI 95% -0.02 – 0.07)] and ‘weak’ agreement [Spearman: *r* = 0.21, *p* = 0.47 and Pearson: *r* = 0.06, *p* = 0.84; Bland Altman lower (-3367) and upper (-1072) limits of agreement all unacceptable for Cr_S_/Cr_DBS_. See Table 1 and Figure 2.

**TABLE 1:** Statistical analysis of Creatine (Cr) and creatinine (Crt) concentrations.

	Mean (SD)		T-Test	Absolute Agreement		Relative Agreement			Correlation			
									Spearman		Pearson	
	SERUM	DBS	p-Value (ES)	SEM	CV(%)	ICC	CI 95%	p-value	r	p-value	r	p-value
**Cr**	691.8 (165.2)	2911 (571.4)	**< 0.001** (5.12)	171.6	27%	0.002	-0.02–0.07	0.46	0.21	0.47	0.06	0.84
**Crt**	813.6 (102.4)	812.4 (108.1)	0.96 (0.01)	88.7	10.7%	0.57	0.06–0.84	0.02	0.77	0.001	0.56	0.04

*ES* – Hedges´*g*; DBS – dried blood spot; SD – standard deviation (μmol/L); SEM – standard error measurements (μmol/L); CV – coefficient of variation; ICC – intraclass correlation coefficient; CI – 95% Confidence Interval; Correlations interpretation: non-important (0 ≤ *r* ≤ 0.20); weak (0.20 < *r* ≤ 0.50); strong (0.50 < *r* ≤ 0.70); very strong (r > 0.70); Hedges´*g* interpretation: trivial (g < 0.10), small (0.10 ≤ g ≤ 0.29), moderate (0.30 ≤ *g* ≤ 0.49), large (0.50 ≤ *g* ≤ 0.69), very large (0.70 ≤ *g* ≤ 0.89) or extremely large (*g* ≥ 0.90); ICC interpretation: small (ICC < 0.50); moderate (0.5 < ICC < 0.75); good (0.75 < ICC < 0.90); or excellent (ICC > 0.90).

## DISCUSSION

Utilizing DBS tissue handling for Crt (Crt_DBS_) demonstrated acceptable agreement, error, and variance (SEM, CV, ICC, 95% CI, Spearman and Person Correlations and Bland Altman) with the established Crt_S_ method (FIA – MS); conversely, Cr_DBS_/Cr_S_ did not. Consequently, the stated hypothesis is accepted only for Crt_S_/Crt_DBS_. The conflicting results between Crt and Cr are likely related to the ‘Matrix Effect’ [13]. ‘Matrix Effect’ is related to the effect on the biological matrix in which analytes are dispersed. The biological matrix contains a higher Cr concentration within the erythrocytes than Crt concentration. Crt flux between the erythrocytes and media is maintained through passive transport to steady-state equilibrium, whereas the active transport between erythrocytes and plasma maintained the gradient of Cr [14]. The drying process of DBS unbalanced the equilibrium of Cr in erythrocytes and media, increasing the transport to media. As a consequence, an overestimation in Cr_DBS_ was observed, but not in Cr_S_ or Crt_S_ [13, 14].

The data demonstrates some utility regarding the practically compatible use of DBS tissue handling, albeit only for Crt. Specific practice facing advantages of DBS include: (i) stability of the sample under ambient conditions for long periods without deterioration in sample accuracy and (ii) that the sample can be transported in envelopes without the need for refrigeration [6]. Additionally, a phlebotomist is not required [8] and thus athlete discomfort and inconvenience are reduced (as is the volume of blood required, and phlebitis and cross infection risk) [8]. Finally, the sample stability offered by DBS tissue handling would be facilitative of multiple participants and time-point sample acquisition [e.g. team sports or a group of Olympic athletes undergoing multiple laboratory evaluations in parallel on the same day; a reality when reliant on centralized facilities (with limited time/staff resource) and their synchronization/integration within the complex training, travel and competition schedules of many athletes (including Olympic)].

This pilot study has shown utility for one target blood-borne biomarker associated with the OR/OTS in Olympic athletes. Proteomic studies utilizing DBS tissue handling are purported in the field mainly linked to immune response and acute phase proteins in OR/ORTS [5, 15]. The limitations of this study (sample size and homogeneity) reinforce the need for further germane field-based DBS data whilst controlling the likely confounding effect of serum capillary blood and the “matrix effect”. A larger and varied professional athlete sample, in addition to a variety of biomarkers and proteomics studies (i.e., those that will evidence-inform athlete preparedness decisions) are required.

### Practical applications

DBS tissue handling has promise and some utility within the sports.

## CONCLUSIONS

This pilot study revealed that Crt_S_ and Crt_DBS_ methods have acceptable agreement, error and variance (Cr_S_ and Cr_DBS_ did not).
